# Different phenotypes of hypertension and associated cardiovascular and all-cause mortality: a systematic review and meta-analysis

**DOI:** 10.1186/s43044-024-00597-w

**Published:** 2024-12-26

**Authors:** Jay Tewari, Khalid Ahmad Qidwai, Shubhajeet Roy, Mehul Saxena, Anadika Rana, Ajoy Tewari, Vineeta Tewari, Anuj Maheshwari

**Affiliations:** 1 King George’s Medical University, Lucknow, India; 2 Department of Internal Medicine, HIND Institute of Medical Sciences, Barabanki, India; 3 Department of Anatomy, Era’s Lucknow Medical College and Hospital, Lucknow, India

**Keywords:** White coat hypertension, Masked hypertension, Cardiovascular mortality, All-cause mortality

## Abstract

**Background:**

Hypertension is a leading cause of premature mortality and morbidity. Recent guidelines advocate for out-of-office blood pressure monitoring, including ambulatory and home BP monitoring, to better identify hypertension phenotypes like masked hypertension, white coat hypertension, and sustained hypertension. However, clinical inertia persists due to a lack of robust evidence on the effectiveness of screening these phenotypes and their association with cardiovascular and all-cause mortality. This systematic review and meta-analysis aims to evaluate the relationship between various hypertension phenotypes and future cardiovascular events and all-cause mortality to support the broader implementation of out-of-office BP monitoring.

**Main body:**

Following PRISMA, Cochrane, and MOOSE guidelines, we conducted a comprehensive search in Pubmed, OvidSP, and Cochrane Central databases up to October 17, 2023. Eligible studies reported associations between hypertension phenotypes and cardiovascular or all-cause mortality, with normotension as the reference group. Hazard ratios with 95% confidence intervals (CIs) were pooled using random-effects models. Eight studies with 15,327 participants were included. Masked hypertension was associated with increased cardiovascular mortality (pooled HR 2.05, 95% CI 1.69–2.48). Sustained hypertension also showed a higher risk (pooled HR 2.42, 95% CI 2.12–2.76). WCH did not significantly increase cardiovascular mortality risk (pooled HR 1.18, 95% CI 0.98–1.42). For all-cause mortality, neither masked hypertension (pooled HR 2.10, 95% CI 0.91–4.88) nor white coat hypertension (pooled HR 1.96, 95% CI 0.71–5.42) showed significant increases.

**Conclusion:**

Masked hypertension and sustained hypertension are linked to higher cardiovascular mortality compared to normotension, highlighting the importance of out-of-office BP monitoring to identify and manage high-risk phenotypes effectively. Further high-quality studies are needed to generalize these findings and support policy changes.

**Supplementary Information:**

The online version contains supplementary material available at 10.1186/s43044-024-00597-w.

## Background

Globally, hypertension is the primary preventable cause of premature mortality and morbidity [1]. The most common way to diagnose hypertension is through blood pressure measurements in the office. Conversely, recent guidelines for the diagnosis and management of hypertension firmly advocate for BP monitoring outside the office, including ambulatory BP monitoring (ABPM) and self-BP monitoring (SBPM) or home BP monitoring (HBPM) [2]. Several blood pressure phenotypes with varying prognostic implications for long-term cardiovascular risk have been identified as a result of the greater implementation of blood pressure monitoring out-of-office in recent years [3]. These blood pressure phenotypes are difficult to diagnose without the readings of both in the office and out-of-office measurements. They comprise sustained normotension, which is defined as normal in the office and outside the office blood pressure in people who are not taking antihypertensive medication; controlled hypertension, which is defined as normal in the office blood pressure and outside the office blood pressure in people taking antihypertensive medication; masked hypertension (MH), which is defined as normal in the office blood pressure but elevated outside the office; white coat hypertension (WCH); increased in the office but normal outside the office blood pressure, which is defined as WCH in individuals that are not on any antihypertensive medications, and as white coat effect (WCE) or white coat uncontrolled hypertension in people taking antihypertensive medication; and uncontrolled hypertension or sustained hypertension, which is elevated in the office and outside the office blood pressure readings [4].

There has been a clinical inertia for adopting this outside the office BP monitoring, despite guideline recommendations, which is most probably due to various patient, provider, and policy-related hurdles. A significant obstacle in obtaining outside the office blood pressure readings is the lack of evidence surrounding the effectiveness of screening for the various phenotypes of hypertension [5]. The studies done for quantifying the relationship between the various phenotypes of hypertension and associated cardiovascular and all-cause mortality have shown inconsistent results [4].

Our goal in conducting this systematic review and meta-analysis (SRMA) is to fully evaluate the relationship between future cardiovascular events and all-cause mortality in the various phenotypes of hypertension. These data may encourage BP monitoring outside of the office to be more broadly recognized and accepted as the standard of care and may help shape policy changes that increase funding and support BP monitoring outside of the office.

## Methods

The Preferred Reporting Items for Systematic Reviews and Meta-Analyses (PRISMA) criteria were adhered to, and the Cochrane Handbook for Systematic Reviews of Interventions principles were followed in this meta-analysis [6]. The Meta-analysis of Observational Studies in Epidemiology (MOOSE) guidelines were followed [7]. The study protocol is registered with PROSPERO under registration number CRD42023473231.

### Study selection criteria

The following PICOS criteria were used to identify and select studies for this meta-analysis: population (P) of interest was people with white coat hypertension, masked hypertension, or sustained hypertension; intervention (I) was not applicable; controls (C) were normotensive people; and the outcomes (O) were cardiovascular mortality, all-cause mortality.

Only publications in the English language that (1) reported associations between different phenotypes of hypertension and cardiovascular mortality, or all-cause mortality, and (2) included a reference group of people with normotension or controlled hypertension, were eligible for inclusion. To ascertain eligibility, two investigators (JT and KAQ) independently screened abstracts and evaluated full texts. A third reviewer resolved any discrepancies (SR).

### Search strategy

An electronic search was undertaken in Pubmed, OvidSP, and Cochrane Central databases from inception till 17 October 2023 using appropriate MeSH terms. The search strategy has been included in the supplementary file 1.

### Data extraction

Using standardized forms, two investigators (JT and KAQ) separately extracted data from every eligible publication. Cohort name, year of publication, type, and duration of out-of-office blood pressure measurement, the total number of study participants, number of participants with a history of diabetes, number of current smokers and male sex, mean age, body mass index, length of follow-up, and type of outcome (cardiovascular mortality or all-cause mortality) were among the extracted data. All the included studies utilized the ACC/AHA criteria for classifying the subjects into various phenotypes of hypertension, i.e., the thresholds were 140/90 mmHg for office BP and 135/85 mmHg for home BP. Any discrepancies were resolved by a third reviewer (AR).

### Risk of bias assessment

Three authors (JT, KAQ, and AT) independently assessed the risk of bias in the included study using the Risk Of Bias In Non-randomized Studies—of Exposures (ROBINS-E) tool [8]. The studies were judged under seven domains. The risk of bias (ROB) table and graph were generated using the Robvis tool [9].

### Measures of treatment effect

Cardiovascular mortality and all-cause mortality were represented as Hazard ratios (HRs) with 95% confidence intervals (CIs). The data were pooled using pooled logarithm hazard ratios using random-effects inverse-variance models, with profile likelihood estimation [10]. The analysis was done on Revman 5.4 [11].

### Heterogeneity assessment

The τ^2^ and *I*^2^ tests were also used to assess heterogeneity. Significant heterogeneity is present when the *P*-value is less than 0.5. The significance of *I*^2^ values depended on the strength of the evidence supporting heterogeneity and the direction and magnitude of treatment effects.

### Data synthesis

A random-effects model was used to pool the data to analyze the outcomes, which were reported as 95% confidence intervals (95% CIs). Using Revman 5.4, forest plots were created. Statistical significance was attained when the *P*-value was less than 0.05. The hazard ratios were pooled using logarithm hazard ratios using inverse-variance models [10].

## Results

### Study selection and characteristics

**Fig. 1:**
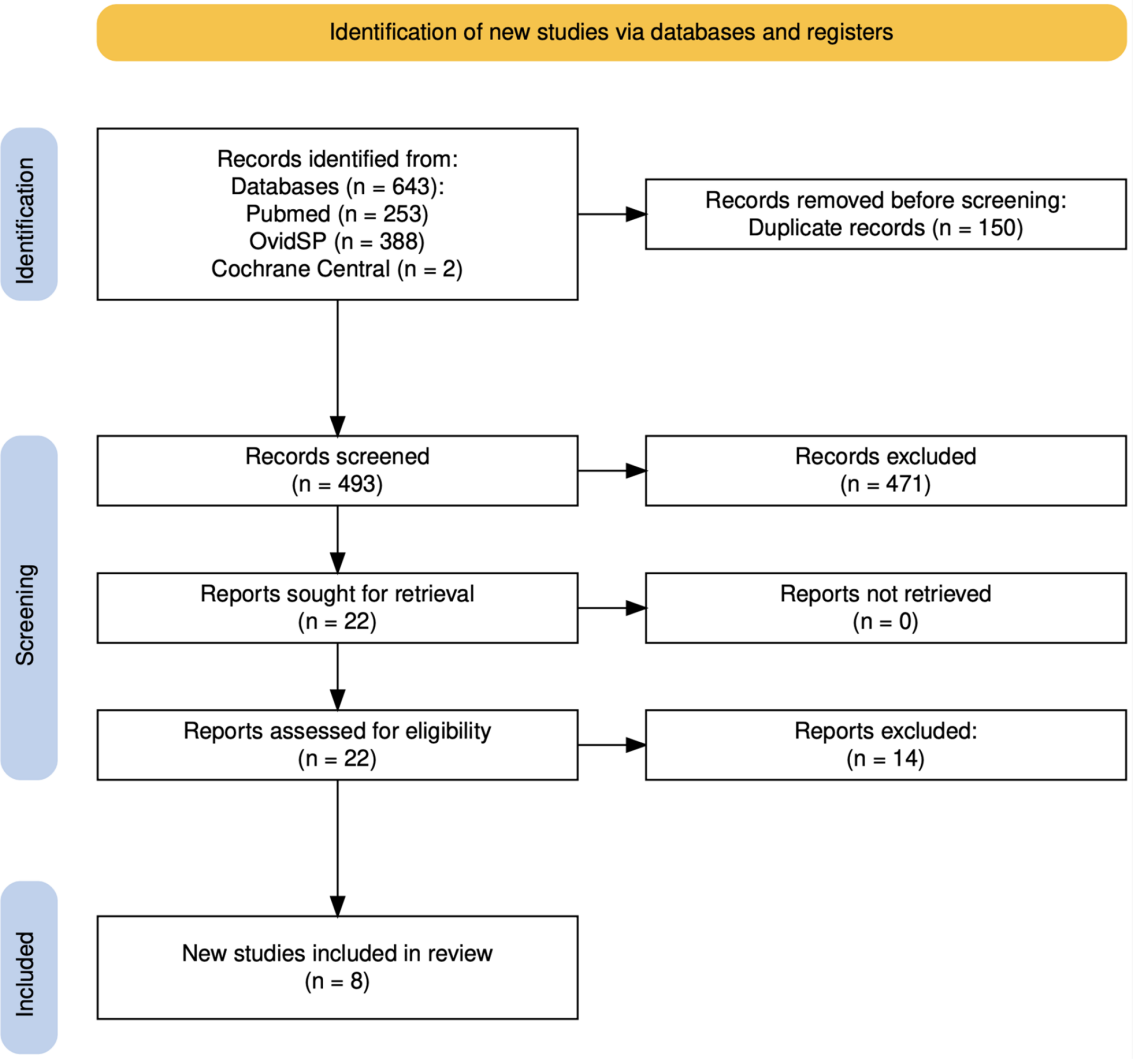
Study selection and inclusion flowchart

Following our initial search (from inception till 17 October 2023), we found 643 articles (Pubmed-253; OvidSP-388; and Cochrane Central-2). After de-duplication (150 duplicates) and screening the articles based on title, abstract, and finally the main text, we finally included 8 studies in the quantitative analysis. The PRISMA study flowchart is shown in Fig. 1 [12]. The characteristics of the included studies and the demographic characteristics of the included participants are shown in Tables 1 and 2, respectively.

**Table 1:** Characteristics of eligible studies

Study	Year of study	Country	Cohort	Type of measurement	Study duration	Total number of participants
Bobrie et al., [13]	2004	France	SHEAF	Home BP	3 Years	4939
Hänninen et al., [14]	2012	Finland	Finn-Home	Home BP	7.5 Years	2046
Hermida et al. [15]	2012	Spain	MAPEC	48-h ABPM	5.6 Years	3344
Minutolo et al., [16]	2014	Italy	Italy-CKD	24-h ABPM	5.2 Years	512
Pereira et al. [17]	2020	Brazil	CKD Cohort	24-h ABPM	8 Years	367
Pierdomenico et al. [18]	2017	Italy	Chieti-Pescara	24-h ABPM	9.1 Years	1191
Satoh et al. [19]	2015	Japan	Ohasama	24-h ABPM and Home BP	17.1 Years	1464
Satoh et al., 2 [20]	2018	Japan	Ohasama	24-h ABPM and Home BP	17.1 Years	1464

**Table 2:** Baseline participant characteristics of included studies

Study	Males (%)	Mean age	Diabetes (%)	Smokers (%)	Mean BMI, kg/m^2^
Bobrie et al., [13]	49	70	15	8	NR
Hänninen, et al., [14]	46	56	6	19	27.4
Hermida et al. [15]	51	53	20	15	29.8
Minutolo et al., [16]	57	64	34	22	28.9
Pereira et al. [17]	44	60	NR	NR	NR
Pierdomenico et al. [18]	42	68	12	12	27.9
Satoh et al. [19]	32	61	14	15	23.4
Satoh et al., 2 [20]	32	61	14	15	23.4

*NR* Not reported

### Risk of bias assessment

**Fig. 2:**
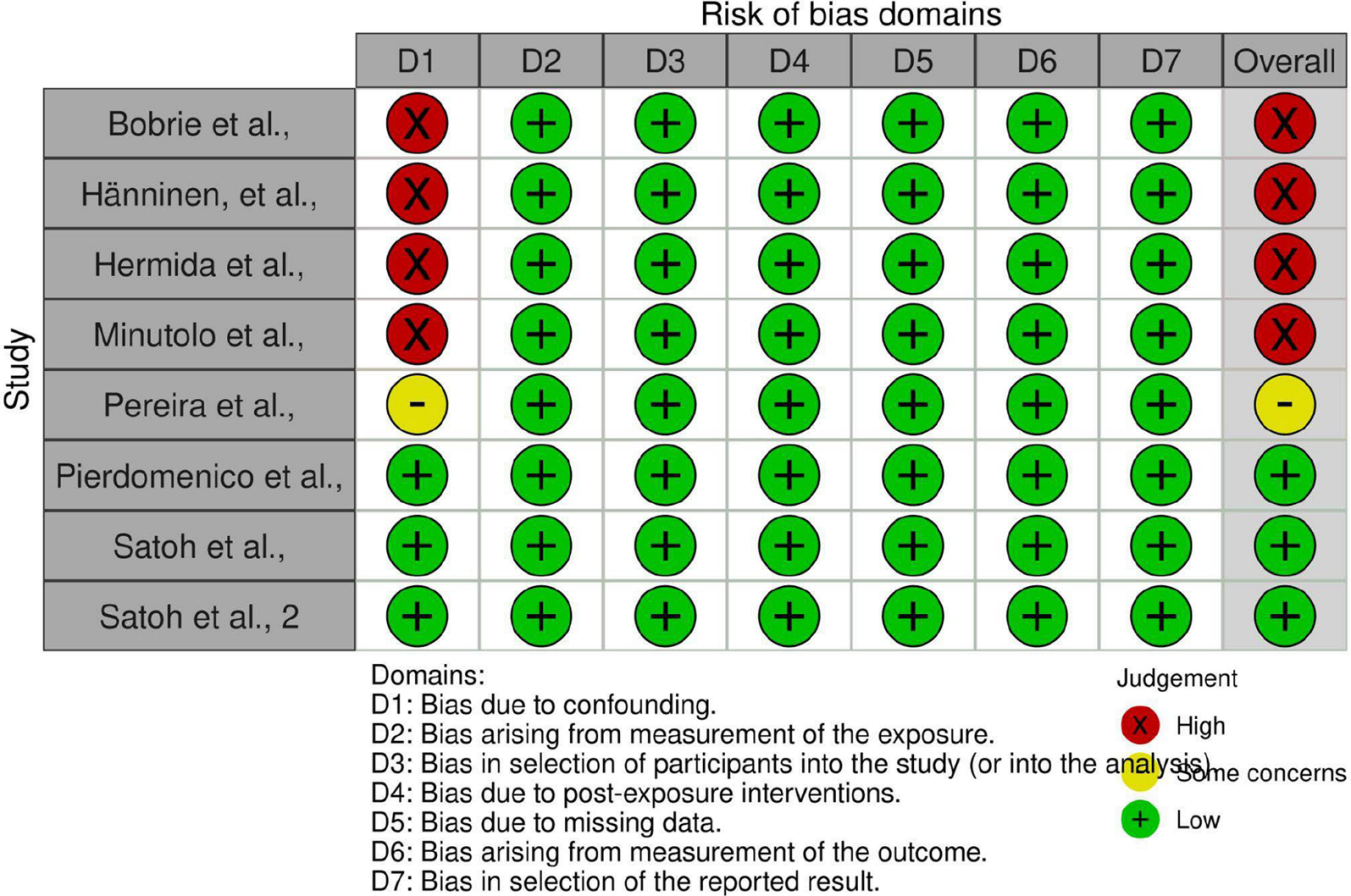
Risk of bias table. *Legend*: References: [13–20]

**Fig. 3:**
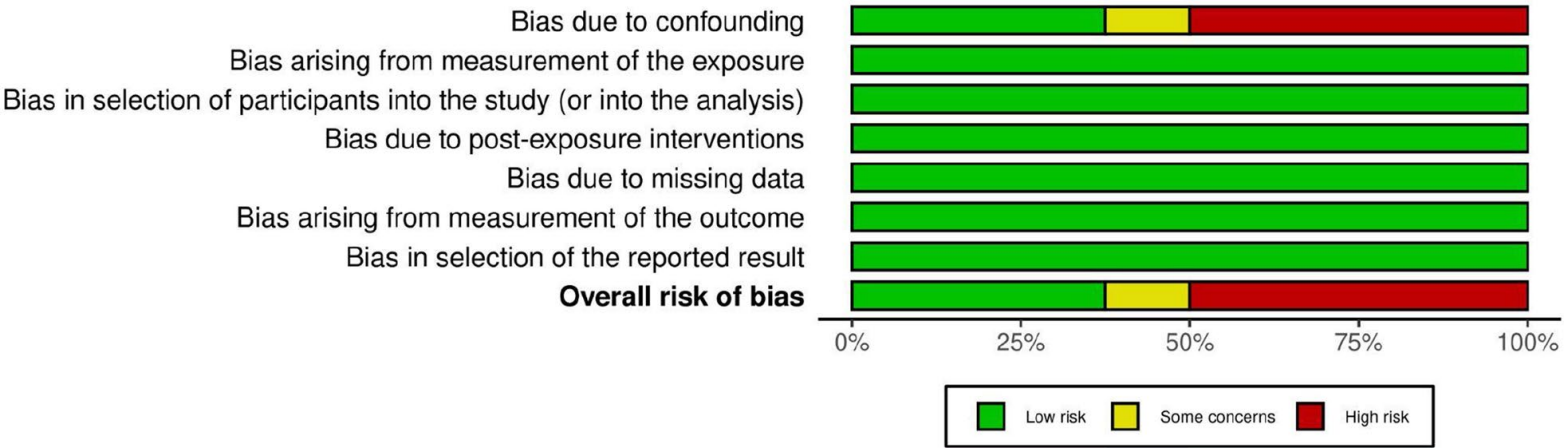
Risk of bias summary plot

The risk of bias assessment summary has been shown in the ROB table and ROB summary plot in Figs. 2 and 3, respectively.

### Cardiovascular mortality

#### Masked hypertension versus normotension

**Fig. 4:**
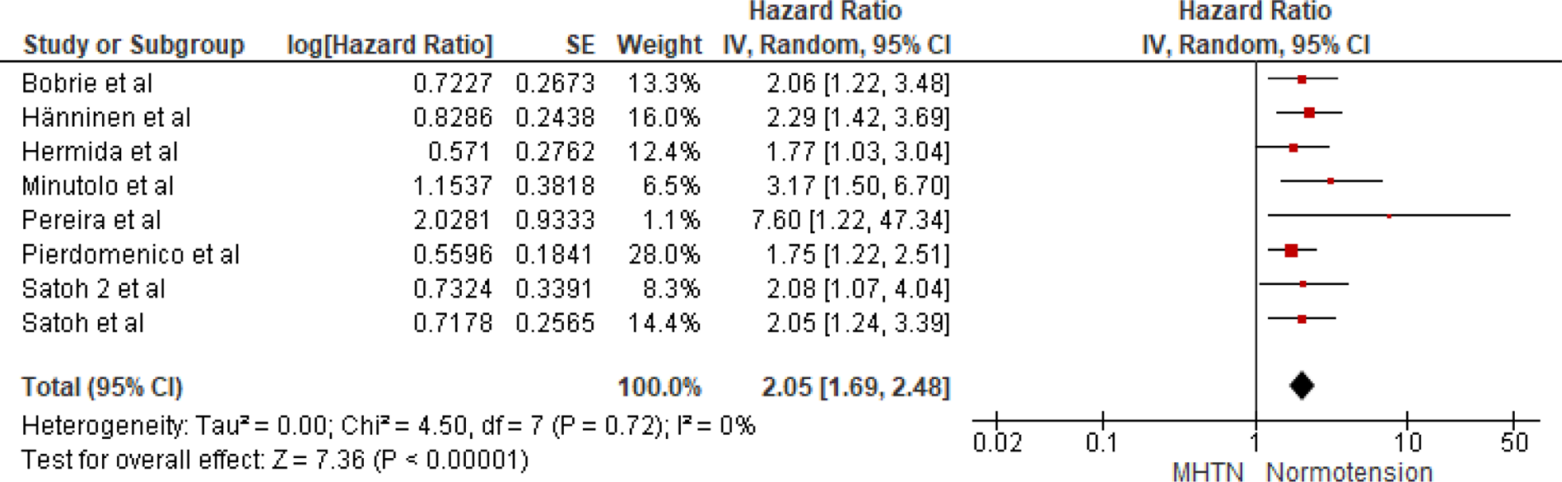
Forest plot for cardiovascular mortality; comparing masked hypertension and normotension. *Legend*: References: [13–20]

Eight studies were included in the analysis of cardiovascular mortality in people with masked hypertension versus normotension. The pooled HR was 2.05 with a 95% CI of 1.69–2.48. The overall effect was significant [*Z* = 7.36 (*P* < 0.00001)]. Insignificant heterogeneity was seen (*P* = 0.72). The forest plot is shown in Fig. 4.

#### White coat hypertension versus normotension

**Fig. 5:**
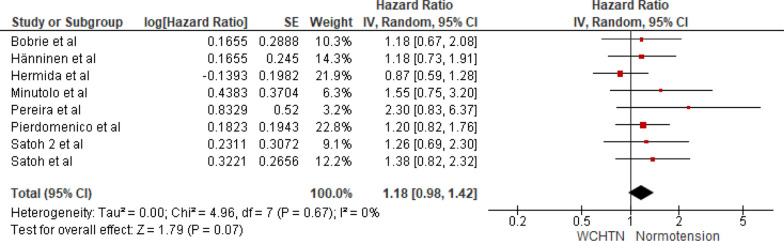
Forest plot for cardiovascular mortality; comparing white coat hypertension and normotension. *Legend*: References: [13–20]

Eight studies were included in the analysis of cardiovascular mortality in people with white coat hypertension versus normotension. The pooled HR was 1.18 with a 95% CI of 0.98–1.42. The overall effect was insignificant [*Z* = 1.79 (*P* = 0.07)]. Insignificant heterogeneity was seen (*P* = 0.72). The forest plot is shown in Fig. 5.

#### Sustained hypertension versus normotension

**Fig. 6:**
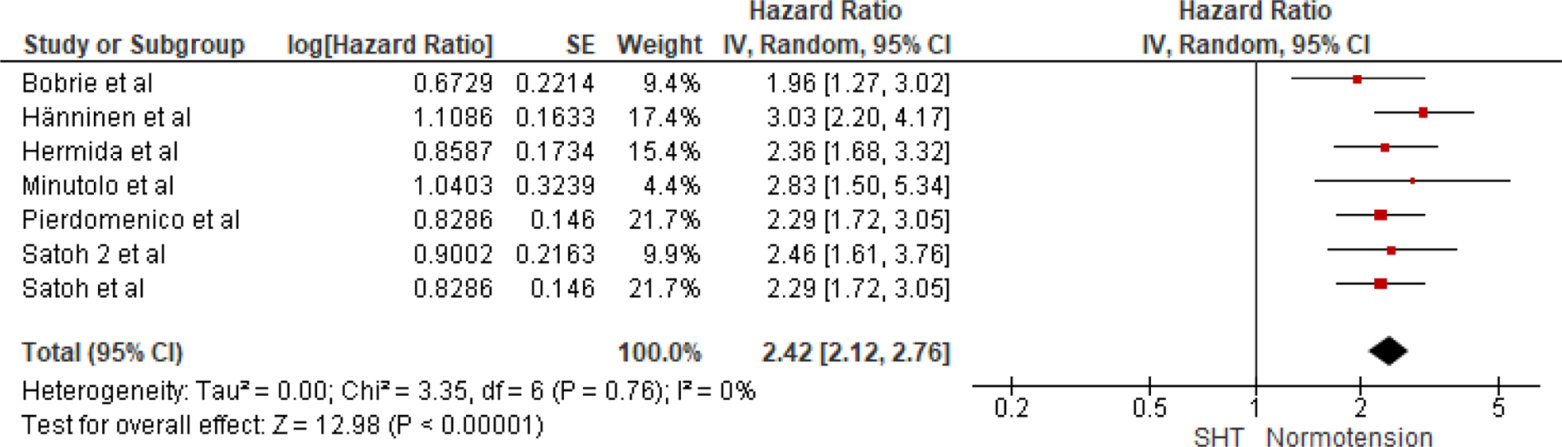
Forest plot for cardiovascular mortality; comparing sustained hypertension and normotension. *Legend*: References: [13–16, 18–20]

Seven studies were included in the analysis of cardiovascular mortality in people with sustained hypertension versus normotension. The pooled HR was 2.42 with a 95% CI of 2.12–2.76. The overall effect was significant [*Z* = 12.98 (*P* < 0.00001)]. Insignificant heterogeneity was seen (*P* = 0.76). The forest plot is shown in Fig. 6.

#### Masked hypertension versus white coat hypertension

**Fig. 7:**
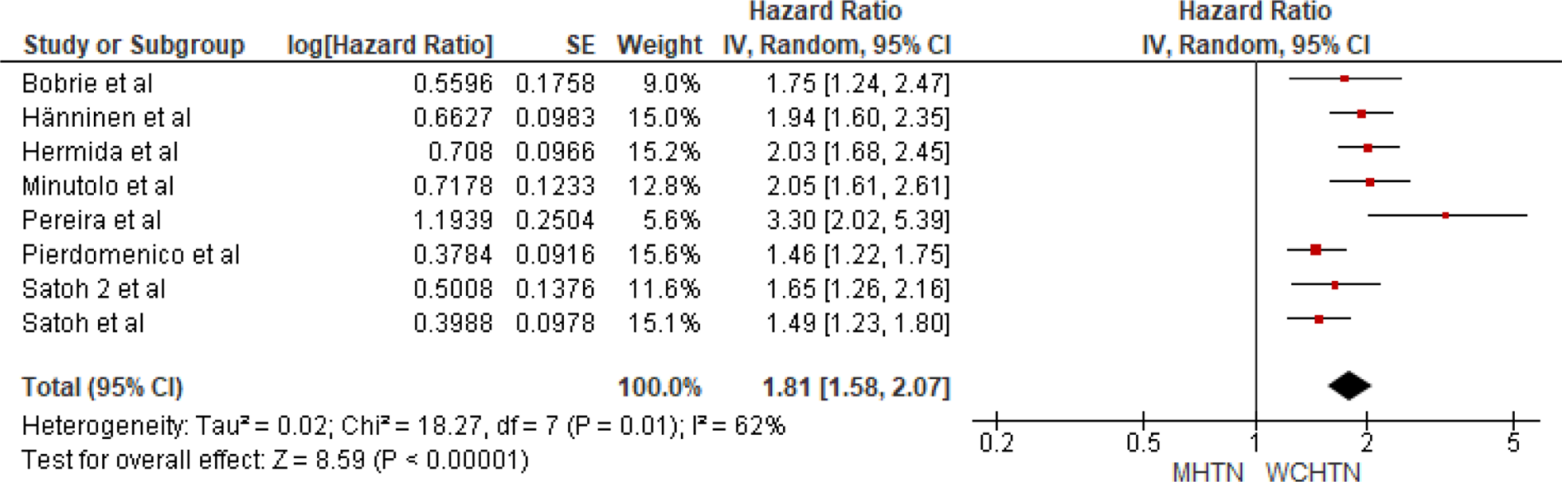
Forest plot for cardiovascular mortality; comparing masked hypertension and white coat hypertension. *Legend*: References: [13–20]

Eight studies were included in the analysis of cardiovascular mortality in people with masked hypertension versus white coat hypertension. The pooled HR was 1.81 with a 95% CI of 1.81–2.07. The overall effect was significant [*Z* = 8.59 (*P* < 0.00001)]. Significant heterogeneity was seen (*P* = 0.01). The forest plot is shown in Fig. 7.

#### Sustained hypertension versus masked hypertension

**Fig. 8:**
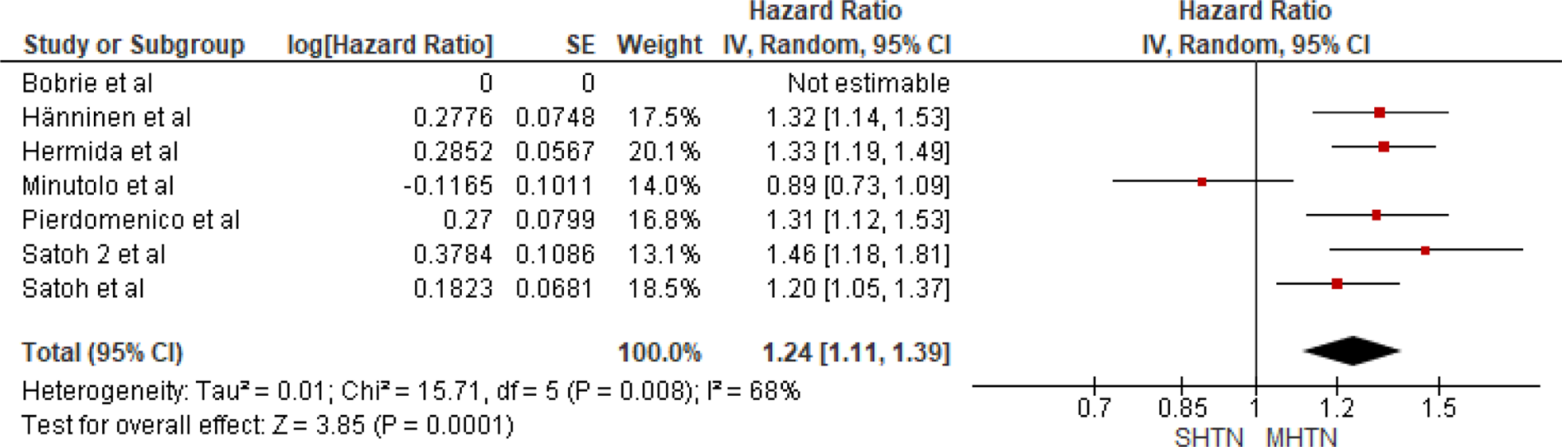
Forest plot for cardiovascular mortality; comparing sustained hypertension and masked hypertension. *Legend*: References: [13–16, 18–20]

Seven studies were included in the analysis of cardiovascular mortality in people with sustained hypertension versus masked hypertension. The pooled HR was 1.24 with a 95% CI of 1.11–1.39. The overall effect was significant [*Z* = 3.85 (*P* = 0.0001)]. Insignificant heterogeneity was seen (*P* = 0.008). The forest plot is shown in Fig. 8.

#### Sustained hypertension versus white coat hypertension

**Fig. 9:**
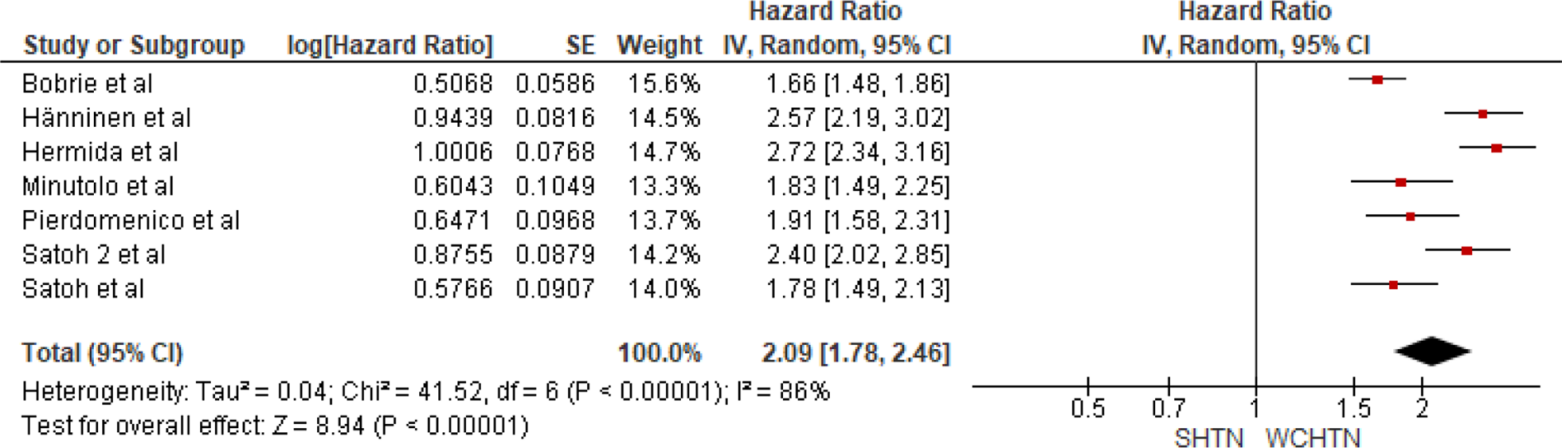
Forest plot for cardiovascular mortality; comparing sustained hypertension and white coat hypertension. *Legend*: References: [13–16, 18–20]

Seven studies were included in the analysis of cardiovascular mortality in people with sustained hypertension versus white coat hypertension. The pooled HR was 2.09 with a 95% CI of 1.78–2.46. The overall effect was significant [*Z* = 8.94 (*P* = 0.0001)]. Insignificant heterogeneity was seen (*P* < 0.00001). The forest plot is shown in Fig. 9.

### All-cause mortality

#### Masked hypertension versus normotension

**Fig. 10:**
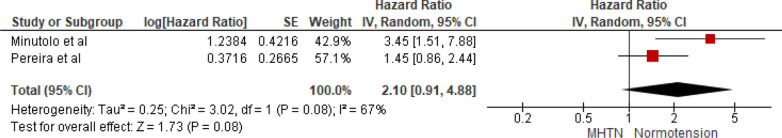
Forest plot for all-cause mortality; comparing masked hypertension and normotension. *Legend*: References: [16, 17]

Two studies were included in the analysis of all-cause mortality in people with masked hypertension versus normotension. The pooled HR was 2.10 with a 95% CI of 0.91–4.88. The overall effect was insignificant [*Z* = 1.73 (*P* = 0.08)]. Insignificant heterogeneity was seen (*P* = 0.08). The forest plot is shown in Fig. 10.

#### White coat hypertension versus normotension

**Fig. 11:**
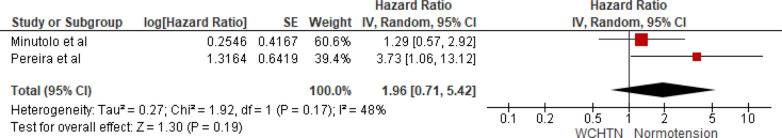
Forest plot for all-cause mortality; comparing white coat hypertension and normotension. *Legend*: References: [16, 17]

Two studies were included in the analysis of all-cause mortality in people with white coat hypertension versus normotension. The pooled HR was 1.96 with a 95% CI of 0.71–5.42. The overall effect was insignificant [*Z* = 1.30 (*P* = 0.19)]. Insignificant heterogeneity was seen (*P* = 0.17). The forest plot is shown in Fig. 11.

## Discussion

The present SRMA includes eight studies with a total of 15,327 participants, who underwent in-office BP measurement and home BP and/or ambulatory BP measurement. In this meta-analysis, we found out that masked hypertension is associated with a significant rise in cardiovascular mortality when compared to normotensive people, with a pooled hazard ratio of 2.05 (95% CI: 1.69–2.48, *P* < 0.00001) and an insignificant heterogeneity (*P* = 0.72). Likewise, sustained hypertension also presents a significantly higher risk, with a pooled HR of 2.42 (95% CI: 2.12–2.76, *P* < 0.00001) and an insignificant heterogeneity (*P* = 0.76). On the other hand, White Coat Hypertension does not significantly increase cardiovascular mortality risk compared to normotension, with a pooled HR of 1.18 (95% CI: 0.98–1.42, *P* = 0.07) and insignificant heterogeneity (*P* = 0.72).

Upon comparing masked hypertension to white coat hypertension, the former shows a significantly higher cardiovascular mortality risk with a pooled HR of 1.81 (95% CI: 1.81–2.07, *P* < 0.00001) and significant heterogeneity (*P* = 0.01). We can hypothesize that this may be because masked hypertension often remains underdiagnosed compared to white coat hypertension. Consequently, timely measures to manage hypertension might not be implemented. Sustained hypertension shows an increased risk of cardiovascular mortality compared with masked hypertension and white coat hypertension, with results being significant with insignificant heterogeneity.

For all-cause mortality, masked hypertension does not show a statistically significant increase in risk compared to normotension, with a pooled HR of 2.10 (95% CI: 0.91–4.88, *P* = 0.08) and insignificant heterogeneity (*P* = 0.08). Similarly, white coat hypertension also does not show a significant increase in all-cause mortality risk compared to normotension, with a pooled HR of 1.96 (95% CI: 0.71–5.42, *P* = 0.19) and insignificant heterogeneity (*P* = 0.17).

We realize that many inconsistencies in findings among research may be explained by variations in study design characteristics. We notice that the older age-group had a greater risk of cardiovascular mortality as compared to the younger age-group. These findings align with the detailed subgroup analyses conducted by Franklin et al., [21]. This may be because such events in younger age-groups require a longer follow-up period. Additionally, shorter follow-up durations attenuated the risk of mortality, whereas longer follow-up studies showed a higher incidence of cardiovascular mortality. This could be due to two reasons: shorter follow-up periods do not account for events occurring later, and white coat or masked hypertension progresses to sustained hypertension in longer follow-up studies.

Upon literature review, we found SRMAs focused on individual phenotypes of hypertension, but few have compared different phenotypes. Our study provides the most updated SRMA comparing various hypertension phenotypes and their association with cardiovascular and all-cause mortality. Unlike most previous SRMAs that utilized fixed-effect models, we employed a random-effects model, which sufficiently accounts for the variations in study design and participant characteristics observed across studies [22, 23]. This SRMA is unique as it directly compares different phenotypes of hypertension. The study was conducted in a standardized manner with rigorous quality control measures. Additionally, the individual studies included in our SRMA are of high quality and have a relatively minimal risk of bias. Moreover, most of the results from our meta-analyses exhibit relatively low heterogeneity.

Our SRMA included only those studies that reported associations between different phenotypes of hypertension and cardiovascular mortality or all-cause mortality. Therefore, the number of studies focusing on individual phenotypes' associations with these outcomes is fewer compared to previous SRMAs. The present study includes only eight studies; a larger number of studies would have produced more robust results. The available studies have been conducted in limited geographical and demographic settings, and more studies would have made the results more generalizable. Therefore, there remains a need for high-quality studies like those included in this SRMA.

## Conclusion

Overall, our analysis shows that masked hypertension and sustained hypertension are associated with significantly higher cardiovascular mortality compared to normotension, while white coat hypertension although associated with worse outcomes was inferior in comparison to both the former phenotypes. Additionally, sustained hypertension poses a higher risk than masked hypertension and white coat hypertension. For all-cause mortality, neither masked hypertension nor white coat hypertension shows a statistically significant increased risk compared to normotension.

## Supplementary Information

Additional file1 (DOCX 7 KB)

## Data Availability

The data can be obtained from the corresponding author upon reasonable request by contacting them through their email ID.

## Abbreviations

BP: Blood pressure
ABPM: Ambulatory blood pressure monitoring
HBPM: Home blood pressure monitoring
MH: Masked hypertension
WCH: White coat hypertension
SRMA: Systematic review and meta-analysis
HRs: Hazard ratios
CIs: Confidence intervals
SBPM: Self-blood pressure monitoring
WCE: White coat effect
PRISMA: Preferred Reporting Items for Systematic Reviews and Meta-Analyses
ROBINS- E: Risk Of Bias In Non-randomized Studies—of Exposures
ROB: Risk of bias

## References

[1] GBD (2017) risk factor collaborators: global, regional, and national comparative risk assessment of 84 behavioural, environmental and occupational, and metabolic risks or clusters of risks for 195 countries and territories, 1990–2017: a systematic analysis for the global burden of disease Study 2017. Lancet Lond Engl 2018(392):1923–1994. 10.1016/S0140-6736(18)32225-6 10.1016/S0140-6736(18)32225-6 PMC6227755 30496105

[2] Whelton PK, Carey RM, Aronow WS et al (1979) 2017 ACC/AHA/AAPA/ABC/ACPM/AGS/APhA/ASH/ASPC/NMA/PCNA guideline for the prevention, detection, evaluation, and management of high blood pressure in adults: a report of the American college of cardiology/American heart association task force on clinical practice guidelines. Hypertens Dallas Tex 2018(71):e13-115. 10.1161/HYP.0000000000000065 10.1161/HYP.0000000000000065 29133356

[3] Shimbo D, Abdalla M, Falzon L, Townsend RR, Muntner P (2015) Role of ambulatory and home blood pressure monitoring in clinical practice: a narrative review. Ann Intern Med 163:691–700. 10.7326/M15-1270 26457954 10.7326/M15-1270 PMC4638406

[4] Cohen JB, Lotito MJ et al (2019) Cardiovascular events and mortality in white coat hypertension: a systematic review and meta-analysis. Ann Intern Med. 170:853–62. 10.7326/M19-0223 31181575 10.7326/M19-0223 PMC6736754

[5] Kronish IM, Kent S, Moise N et al (2017) Barriers to conducting ambulatory and home blood pressure monitoring during hypertension screening in the United States. J Am Soc Hypertens JASH 11:573–580. 10.1016/j.jash.2017.06.012 28734798 10.1016/j.jash.2017.06.012 PMC5595651

[6] Page MJ, McKenzie JE, Bossuyt PM et al (2021) The PRISMA 2020 statement: an updated guideline for reporting systematic reviews. BMJ 372:n71. 10.1136/bmj.n71 33782057 10.1136/bmj.n71 PMC8005924

[7] Stroup DF, Berlin JA, Morton SC et al (2000) Meta-analysis of observational studies in epidemiology: a proposal for reporting. Meta-analysis Of Observational Studies in Epidemiology (MOOSE) group. JAMA 283:2008–12. 10.1001/jama.283.15.2008 10789670 10.1001/jama.283.15.2008

[8] Higgins JPT, Morgan RL, Rooney AA et al (2024) A tool to assess risk of bias in non-randomized follow-up studies of exposure effects (ROBINS-E). Environ Int 186:108602. 10.1016/j.envint.2024.108602 38555664 10.1016/j.envint.2024.108602 PMC11098530

[9] McGuinness LA, Higgins JPT (2020) Risk-of-bias VISualization (robvis): an R package and Shiny web app for visualizing risk-of-bias assessments. Res Synth Methods 12(1):55–61. 10.1002/jrsm.1411 32336025 10.1002/jrsm.1411

[10] Cornell JE, Mulrow CD, Localio R, Stack CB, Meibohm AR, Guallar E, Goodman SN (2014) Random-effects meta-analysis of inconsistent effects: a time for change. Ann Intern Med 160:267–270. 10.7326/M13-2886 24727843 10.7326/M13-2886

[11] Review Manager (RevMan) [Computer program]. Version 5.4. The Cochrane Collaboration, (2020)

[12] Haddaway NR, Page MJ, Pritchard CC, McGuinness LA (2022) PRISMA2020: an R package and Shiny app for producing PRISMA 2020-compliant flow diagrams, with interactivity for optimised digital transparency and open synthesis. Campbell Syst Rev 18:e1230. 10.1002/cl2.1230 36911350 10.1002/cl2.1230 PMC8958186

[13] Bobrie G, Chatellier G, Genes N et al (2004) Cardiovascular prognosis of ‘masked hypertension’ detected by blood pressure self-measurement in elderly treated hypertensive patients. JAMA 291:1342–1349 15026401 10.1001/jama.291.11.1342

[14] Hanninen M-R, Niiranen T, Puukka P, Johansson J, Jula A (2011) Prognostic significance of masked and white-coat hypertension in the general population: the Finn-home study. J Hypertens 29:e311 10.1097/HJH.0b013e328350a69b 22278146

[15] Hermida RC, Ayala DE, Mojón A, Fernández JR (2012) Sleep-time blood pressure and the prognostic value of isolated-office and masked hypertension. Am J Hypertens 25:297–305. 10.1038/ajh.2011.208 22089106 10.1038/ajh.2011.208

[16] Minutolo R, Gabbai FB, Agarwal R et al (2014) Assessment of achieved clinic and ambulatory blood pressure recordings and outcomes during treatment in hypertensive patients with CKD: a multicenter prospective cohort study. Am J Kidney Dis Off J Natl Kidney Found 64:744–752. 10.1053/j.ajkd.2014.06.014 10.1053/j.ajkd.2014.06.014 25082100

[17] da Pereira Silva H, Bonilha A, Barretti P, Silva R, Burgugi V, dos Santos V, Cuadrado L (2020) White-coat and masked hypertension diagnoses in chronic kidney disease patients. J Clin Hypertens Greenwich Conn 22:1202–7. 10.1111/jch.13924 10.1111/jch.13924 PMC8029747 32608106

[18] Pierdomenico SD, Pierdomenico AM, Coccina F, Porreca E (2017) Prognosis of masked and white coat uncontrolled hypertension detected by ambulatory blood pressure monitoring in elderly treated hypertensive patients. Am J Hypertens 30:1106–1111. 10.1093/ajh/hpx104 29059303 10.1093/ajh/hpx104

[19] Satoh M, Asayama K, Kikuya M et al (1979) Long-term stroke risk due to partial white-coat or masked hypertension based on home and ambulatory blood pressure measurements: the Ohasama study. Hypertens Dallas Tex 2016(67):48–55. 10.1161/HYPERTENSIONAHA.115.06461 10.1161/HYPERTENSIONAHA.115.06461 26527046

[20] Satoh M, Asayama K, Murakami T, Kikuya M, Metoki H, Imai Y, Ohkubo T (2019) Stroke risk due to partial white-coat or masked hypertension based on the ACC/AHA guideline’s blood pressure threshold: the Ohasama study. Hypertens Res Off J Jpn Soc Hypertens 42:120–122. 10.1038/s41440-018-0133-2 10.1038/s41440-018-0133-2 30401908

[21] Franklin SS, Thijs L, Asayama K et al (2016) The cardiovascular risk of white-coat hypertension. J Am Coll Cardiol 68:2033–2043. 10.1016/j.jacc.2016.08.035 27810041 10.1016/j.jacc.2016.08.035

[22] Huang Y, Huang W, Mai W et al (2017) White-coat hypertension is a risk factor for cardiovascular diseases and total mortality. J Hypertens 35:677–688. 10.1097/HJH.0000000000001226 28253216 10.1097/HJH.0000000000001226 PMC5338886

[23] Briasoulis A, Androulakis E, Palla M, Papageorgiou N, Tousoulis D (2016) White-coat hypertension and cardiovascular events: a meta-analysis. J Hypertens 34:593–599. 10.1097/HJH.0000000000000832 26734955 10.1097/HJH.0000000000000832

