# Supplementary material for: Different phenotypes of hypertension and associated cardiovascular and all-cause mortality: a systematic review and meta-analysis
Source: Egypt Heart J. 2024 Dec 26;76:162. doi: 10.1186/s43044-024-00597-w (PMC11671445; doi:10.1186/s43044-024-00597-w)
Supplement: Supplementary file 1 — Additional file1 (DOCX 7 KB) [file 43044_2024_597_MOESM1_ESM.docx]

**Supplementary Material**

("masked hypertension"[MeSH Terms] OR "masked hypertension"[All Fields] OR "masked HTN"[All Fields] OR "MHTN"[All Fields]) AND ("white coat hypertension"[MeSH Terms] OR "white coat hypertension"[All Fields] OR "WCH"[All Fields] OR "WC HTN"[All Fields] OR "WCHTN"[All Fields] OR "white coat HTN"[All Fields]) AND (mortality/ OR death/ OR myocardial ischemia/ OR heart failure/ OR cardiomyopathies/ OR stroke/ OR mortality[tw] OR death[tw] OR cardiovascular[tw] OR stroke[tw] OR myocardial infarct*[tw] OR heart failure[tw] OR cardiomyopathy*[tw] OR cerebrovascular[tw])

252 results 05:39:39

253 16/10/2023

OVID SP

Search KGMU Full-text Journals

Search All Ovid Journals

1 masked hypertension.mp. [mp=ti, ab, tx, ct] 3455

2 white coat hypertension.mp. [mp=ti, ab, tx, ct] 5282

3 cardiovascular mortality.mp. [mp=ti, ab, tx, ct] 54617

4 1 and 2 and 3 388

Search Name:

Date Run: 17/10/2023 17:13:09

Comment:

ID Search Hits

#1 MeSH descriptor: [Masked Hypertension] explode all trees 20

#2 MeSH descriptor: [White Coat Hypertension] explode all trees 35

#3 mortality 118404

#4 #1 AND #2 2
